# Smallpox at the Cape of Good Hope

**Published:** 1859-02

**Authors:** 


*Smallpox at the Cape of Good Hope.*—The schooner Wm. M. Dodge, from Cape Town, Nov. 27th, reports the smallpox and fever raging there with great fatality. The Cape Town Advertiser says, that if proper remedial measures had been adopted, one thousand lives would have been saved in the brief time the epidemic has been raging.

